# Improving the outcomes of sepsis in Brazil: strategies and initiatives

**DOI:** 10.62675/2965-2774.20250313

**Published:** 2025-05-28

**Authors:** Daniela Carla de Souza, Regis Goulart Rosa, Reinaldo Salomão, Flávia Ribeiro Machado

**Affiliations:** 1 Universidade de São Paulo Hospital Universitário São Paulo SP Brazil Hospital Universitário, Universidade de São Paulo - São Paulo (SP), Brazil.; 2 Instituto Latino Americano de Sepse São Paulo SP Brazil Instituto Latino Americano de Sepse - São Paulo (SP), Brazil.; 3 Hospital Moinhos de Vento Porto Alegre RS Brazil Hospital Moinhos de Vento - Porto Alegre (RS), Brazil.; 4 Universidade Federal de São Paulo Escola Paulista de Medicina São Paulo SP Brazil Escola Paulista de Medicina, Universidade Federal de São Paulo - São Paulo (SP), Brazil.

## INTRODUCTION

Sepsis represents a global health problem recognized by the World Health Organization (WHO) as a priority due to its high incidence, morbidity, mortality, and the substantial social and economic burden it imposes. The impact is especially severe in low- and middle-income countries (LMICs), which bear the brunt of the estimated 48 million sepsis cases and 11 million sepsis-related deaths annually.^(1)^ Brazil exemplifies the challenges faced by LMIC, with considerable disparities in income, healthcare access, education, basic sanitation, and technology. Indicators such as low vaccination rates, high prevalence of preventable conditions, and an overburdened public health system highlight the country's vulnerability to sepsis.

Septic patients may present at all levels of the healthcare system and must be recognized early and receive appropriate care wherever they are. Quality management of sepsis requires effective healthcare systems that can provide early recognition and detection of clinical deterioration, timely emergency care, targeted antimicrobial therapy, source control, intensive care management, prevention of complications, and adequate rehabilitation. Thus, an urgently needed global solution will require actions to reduce inequalities, from preventative measures to early recognition and fair treatment for all at-risk people.

The *Instituto Latino Americano de Sepse* (ILAS) was founded in 2004 by a group of dedicated professionals.^(2)^ Throughout its 20-year history, ILAS has worked to raise awareness and reduce the burden and impact of sepsis in Brazil and Latin America, aligned with the WHO resolution and promoting actions in all areas to reduce the burden of sepsis.

## Understanding the challenge

*Instituto Latino Americano de Sepse* encourages and promotes research on sepsis and has made considerable progress in better understanding the burden of sepsis. In recent decades, several impactful articles on sepsis in Brazil have been published in high-impact journals. Initial studies showing high mortality rates^(3-5)^ and costs^(6)^ were followed by more recent ones reinforcing our knowledge of sepsis epidemiology.^(7-9)^ In 2014, the SPREAD ICU, a 1-day point prevalence study in a stratified random sample of Brazilian adult intensive care units (ICUs), reported a prevalence of 30% and an overall mortality rate of 56% among septic patients admitted in ICU.^(7)^ In 2019, a similar study conducted in pediatric ICUs showed prevalence of 25%, with a mortality rate of 20%.^(8)^ Based on these studies, population estimates suggested that more than 450 thousand patients have sepsis treated in the ICU per year, resulting in more than 240 thousand deaths among adults in Brazil. Among children, the estimates were more than 40 thousand cases, with an estimated 8,300 deaths per year. Preventable factors, such as incomplete or unknown vaccination status and healthcare-associated infections, were associated with higher mortality rates in children.^(8)^ In contrast, among adults, the low availability of resources and inadequacy of treatment were independently associated with higher mortality.^(7)^ In another study in a non-random sample of adult Emergency Departments in Brazil, the mortality rate from sepsis was 32%.^(9)^ Notably, allocation patterns were inadequate, with 39.2% of patients remaining in public emergency departments during the whole hospital stay, among whom 55.4% died. In this study, age, severity of illness, healthcare-associated sepsis, and being admitted to a low-resource institution were associated with higher mortality rates. In contrast, admission to accredited institutions was associated with better outcomes. These studies allowed a better understanding of the sepsis burden in our country and showed that modifiable factors, such as resources and quality of care, are associated with reduced mortality. The international recognition of ILAS role is illustrated by having representatives in both adults and pediatrics international guidelines such as the Surviving Sepsis Campaign guidelines^(10-13)^ and also the Pediatric Sepsis Definitions Task Force.^(14)^

## Increasing awareness and education

Previous data has already shown that knowledge about sepsis is limited both among lay people^(15)^ and healthcare workers.^(16)^ Promoting actions to increase awareness about sepsis and its prevention, early recognition, and treatment are key points to change patient outcomes. Over the years, ILAS has engaged hospitals in activities aiming to increase awareness. The launch of the World Sepsis Day by the Global Sepsis Alliance (GSA) in 2012, followed by the WHO acknowledgment in 2017,^(17)^ further increased these activities all around the globe. Following the WHO resolution, regional alliances were established to improve implementation at a local level. *Instituto Latino Americano de Sepse* and other regional societies in Latin America approved the *São Paulo Declaration*. The declaration contains the main requests to the government, legislators, health managers and professionals, and societies. In June 2021, the city of São Paulo approved a law mandating the implementation of sepsis protocol in all public healthcare institutions.

Since the first International Sepsis Forum, which celebrated its 20th edition in 2024,^(18)^ ILAS has strived to converge basic and clinical researchers in the field of sepsis. Brazil has a tradition of excellence in basic research, among others, in Microbiology, Immunology, and Pharmacology, which are closely related to sepsis. In sessions dedicated to translational research, the event brings together basic and clinical researchers in a rich environment to improve the challenge of translating results from bench to bedside and generating bedside-to-bench feedback. This interaction increases our understanding of the pathogenesis of sepsis and envisions new therapeutic targets. The meeting addresses clinical research, bedside management, and quality improvement initiatives in adults and pediatrics. Believing that education is the key to success in reducing the burden of sepsis, ILAS in 2019 launched a free online education program for healthcare professionals with topics related to managing sepsis in newborns, children, and adults. More than ten thousand students and healthcare professionals have already accessed this educational platform and watched these programs.

## Improving recognition, treatment, and survivorship

For the last two decades, ILAS has worked closely with hundreds of public and private institutions to implement initiatives to improve the quality of sepsis care. A study in a network of private hospitals in Brazil, in partnership with the ILAS quality improvement program, demonstrated an association between compliance with sepsis bundle and reduction in mortality.^(19)^ There was a reduction in the total cost per patient, with full compliance with the bundles being associated with a US$5,383 saving per year of quality-adjusted life. Other studies, also coordinated by ILAS, reinforced the association between a reduction in the risk of death with earlier recognition and adherence to care bundles.^(20)^ However, public hospitals face additional challenges to sustain quality improvement initiatives.^(5)^ Possible reasons are the population served by these facilities, lower awareness about sepsis, delays in arrival to the emergency room, lack of adequate resources, lack of ICU beds, lack of healthcare professionals, and staff turnover.

As part of its ongoing commitment to improving sepsis outcomes, ILAS launched *Reabilita Sepse* in 2022, a scientifically grounded educational platform to address the rehabilitation needs of sepsis survivors and their families.^(21)^ This initiative provides evidence-based information on the multifaceted rehabilitation process—encompassing physical, cognitive, and mental health recovery. Recognizing that post-sepsis disabilities are often underdiagnosed yet responsive to intervention, *Reabilita Sepse* seeks to mitigate the long-term impacts of sepsis by closing the educational gap regarding rehabilitation challenges. The program's content is rigorously reviewed and validated by an interdisciplinary team of experts, ensuring that the guidance is scientifically sound, practically applicable, and accessible at no cost to patients and their families.

## Perspectives

In alignment with the WHO Resolution, which emphasizes the need for high-quality data collection — particularly in LMICs — and highlights the importance of education to enhance sepsis prevention, diagnosis, and treatment, we believe that addressing socioeconomic disparities in our country is only one aspect of the solution. It is equally crucial to promote strong political leadership and foster multilateral cooperation that acknowledges the profound impact of sepsis on our region. Strengthening the overall quality of the health system is essential to ensure it can adequately manage septic patients and address the long-term effects of the condition. There is an urgent need to develop more accurate diagnostic tools, implement more potent prevention strategies, and reinforce the role of scientific evidence in quality improvement programs for sepsis care. Moreover, encouraging local research and innovation in sepsis management is vital. The fight against sepsis is a shared responsibility that must engage all sectors of society, and the collective success of these efforts should be celebrated. Our journey is long, but we are already working to win the fight against sepsis in Brazil. We believe that high-quality care with limited resources is possible. ILAS remains committed to its mission of eliminating preventable deaths from sepsis in Brazil.
